# A Physiotherapeutic Approach to a Rare Case of Windswept Deformity in a Male Adolescent

**DOI:** 10.7759/cureus.53350

**Published:** 2024-01-31

**Authors:** Rutuja G Sawalkar, Deepali S Patil, Richa S Gandhi

**Affiliations:** 1 Department of Musculoskeletal Physiotherapy, Ravi Nair Physiotherapy College, Datta Meghe Institute of Higher Education and Research, Wardha, IND

**Keywords:** gait training, physical rehabilitation, primary hyperparathyroidism, genu varus, genu valgus, windswept deformity

## Abstract

Primary hyperparathyroidism (PHPT) can lead to a rare condition in children and adolescents known as windswept deformity. This deformity involves one knee exhibiting an abnormal outward angulation (valgus deformity), while the other knee shows an abnormal inward angulation (varus deformity). This asymmetrical syndrome, resembling the effect of strong winds, gives the impression that the knees are being swept in opposite directions. Various factors, such as structural bone or joint defects, accidents, or underlying disorders, can contribute to the development of windswept deformity. PHPT, a common endocrine condition characterized by elevated levels of parathyroid hormone and blood calcium, is unusual in the pediatric and adolescent populations. It can result in complications like osteoporosis and bone abnormalities, with genu valgus (outward knee angulation) being an exceptionally rare symptom. This case discusses a 19-year-old male who underwent corrective surgery for genu valgus and presented with windswept deformity due to teenage hyperparathyroidism. The case study outlines the physiotherapeutic rehabilitation strategy, emphasizing treatments such as cryotherapy, patellar mobilization, and gait training. Tailored physical therapy rehabilitation plays a crucial role in the postoperative care of patients undergoing corrective osteotomies. The results indicated a significant improvement in muscle strength, an expansion of the range of motion (ROM), and a noticeable enhancement in the individual's functional autonomy following adherence to the postoperative physiotherapy (PT) plan.

## Introduction

The term "windswept deformity" is a medical word that refers to a particular ailment where one knee exhibits an abnormal outward angulation known as valgus deformity and the other knee exhibits an abnormal inward angulation known as varus deformity. The asymmetry of this disease is notable because it gives the appearance that a powerful wind has "swept" the two knees in opposite directions. Several underlying reasons, including structural defects in the bones or joints, injury, or illness, may cause this deformity to develop [1]. Windswept deformity is the term for the condition where the knees are positioned at the two opposite ends of the deformity spectrum in the coronal plane. Both the bone and soft tissue components are deficient at each of these extremes [2,3]. Skeletal dysplasias and metabolic bone diseases are commonly connected to windswept deformities [4].

Primary hyperparathyroidism (PHPT) is a prevalent endocrine disorder marked by elevated levels of calcium in the blood and elevated or inappropriately normal levels of parathyroid hormone in the serum [5,6]. It is an infrequent condition in growing populations [7,8]. PHPT may present with osteoporosis and increased urinary calcium excretion, and it can also manifest as vertebral fractures and changes in bone structures, both of which might occur without noticeable symptoms [5]. Although bone-related problems have been identified as early signs of PHPT in adolescents, the manifestation of genu valgus (outward angulation of the knees) in this context is exceptionally uncommon [9].

Patellar mobilization therapy (PMT) refers to a therapeutic approach focused on the patella. This technique is used in the management of various knee conditions and post surgery [10]. During PMT, physical therapists manipulate the position and movement of the patella to address issues like malalignment, tightness, and restricted motion. Dysfunction in patellar function due to PMT impairment can improve the patient's capability to engage in daily activities and participate in physical or sports-related endeavors, ultimately contributing to an enhanced quality of life [11]. Cold compression therapy enhances pain management and consequently holds the potential for enhancing the range of motion (ROM). The advantages of incorporating cold therapy into the comprehensive care of orthopaedic patients encompass effective pain modulation and reduced swelling, facilitating earlier rehabilitation [12]. This research paper explores a case study of a 19-year-old male patient who has been diagnosed with a windswept deformity caused by PHPT. The main focus of this study is to emphasize the importance of physiotherapy (PT) in managing this condition following post-cannulated cancellous screw fixation of the distal femur.

## Case presentation

Patient information

**Figure 1:**
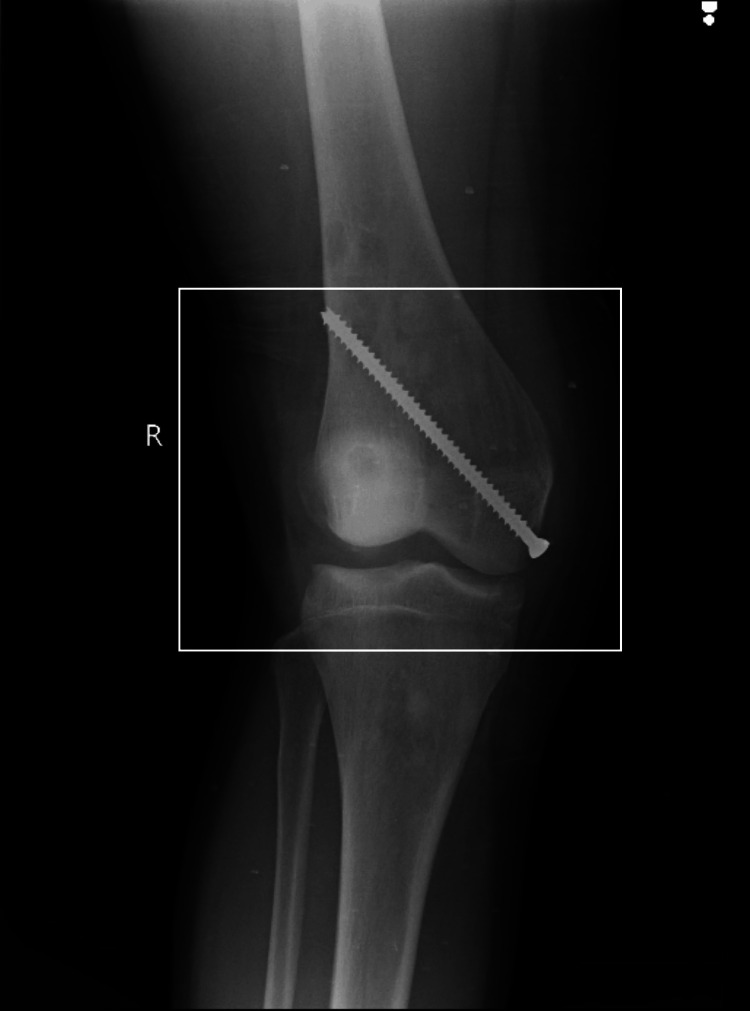
Hemiepiphysiodesis of the right knee with cannulated cancellous screw fixation of the distal femur The square represents hemiepiphysiodesis of the right knee with cannulated cancellous screw fixation of the distal femur

A male aged 19, left-hand dominant, complained of deformities in both lower limbs. He noticed a deformity in his right knee nine months ago. The deformity was present in each knee, in which the right knee bends medially and the left knee bends laterally. Deformity progressed as time passed. Pain in each knee was insidious in onset and progressive, which was 5/10 on the Numerical Pain Rating Scale (NPRS). Walking and movement exacerbated the pain (7/10 on NPRS), which was alleviated during periods of rest and with the aid of medications. The patient went to a private clinic where investigations like X-rays were done and was referred to Acharya Vinoba Bhave Rural Hospital (AVBRH) for surgical management. Diagnostic investigations such as X-rays, magnetic resonance imaging (MRI), and T3 and T4 serum calcium were done. The patient underwent parathyroidectomy for a parathyroid adenoma on June 30, 2023, and soft tissue reconstruction of the right knee and hemiepiphysiodesis of the right knee with cannulated cancellous screw fixation of the distal femur on September 14, 2023 (Figure 1). Rehabilitation through PT began with a customized protocol designed specifically for the patient's needs after surgery.

Clinical findings

Before the commencement of the examination, the patient provided informed consent, and subsequently, a thorough examination was conducted. During the examination, he exhibited cooperation, lethargy, and a clear orientation to person, place, and time. The patient was afebrile, and his hemodynamic status was stable. He was seen in a supine lying posture, with the head elevated to 30°. In addition, he was ectomorphic, with a body mass index (BMI) of 20 kg/m^2^. His speech, vision, and hearing were normal. The ROM of the right lower limb was taken (Table 1). The strength was evaluated through manual muscle testing (MMT) (Table 2).

**Table 1:** Pre-intervention ROM ROM: range of motion

Joints	ROM (right)	ROM (left)
Hip flexion	40°	95°
Hip abduction	30°	25°
Knee flexion	40°	90°

**Table 2:** Pre-intervention muscle strength on the MMT scale according to MRC grading MMT: manual muscle testing; MRC: Medical Research Council; Grade 0: no contraction; Grade 1: flicker of contraction; Grade 2: full range of motion actively in anti-gravity position; Grade 3: full range of motion actively against gravity; Grade 4: full range of motion actively against gravity with minimal resistance; Grade 5: full range of motion actively against gravity with maximal resistance

Muscles	MMT grade (right)	MMT grade (left)
Hip flexors	1/5	3/5
Hip extensors	1/5	3/5
Hip abductors	1/5	3/5
Hip adductors	1/5	3/5
Knee flexors	1/5	3/5
Knee extensors	1/5	3/5

Diagnostic assessment

**Figure 2:**
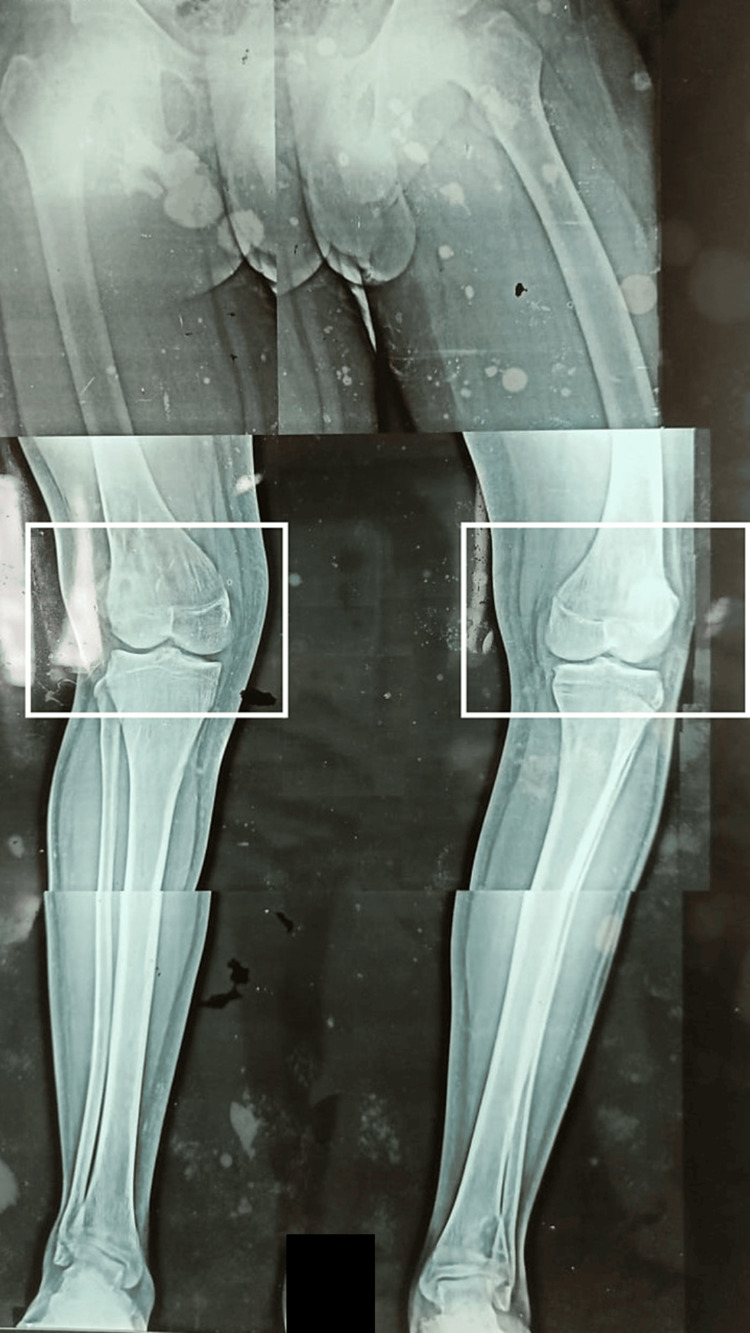
X-ray of bilateral knee joint The rectangle shows genu valgus in the right knee and genu varus in the left knee. The image is a combination of three different X-rays

X-rays were performed, revealing genu valgus in the right knee and genu varus in the left knee, as depicted in Figure 2.

PT intervention

The PT rehabilitation protocol was planned (Table 3).

**Table 3:** Physiotherapy protocol ROM: range of motion; ADLs: activities of daily living; kg: kilogram

Problem identified	Goals	Treatment strategy	Intervention	Progression
Patient and family education	To augment and sustain a patient's favorable outlook toward their treatment regimen, facilitating an expedited recuperation process	The engagement of the therapist with both the patient and their family	The patient, accompanied by their family, received comprehensive explanations about the patient's condition and was informed about the critical role of physiotherapy intervention	The home program was explained
Pain	To relieve pain	Cryotherapy	Icing given for 10-20 minutes	-
Weakness in muscles	To increase the strength of the muscles	Strengthening exercises	Static strengthening exercises for quadriceps, hamstrings, and glutei	Strengthening exercises with 1 kg weighted cuff
			Engaging in strengthening routines using a 0.5 kg weighted cuff	
Decreased knee ROM	To increase the ROM	Graded knee mobilization	Patellar mobilization in medial and lateral and superior and inferior glides	-
Difficulty in ADLs	To facilitate the ADLS	Task-specific training	Functional reach-outs	-
Difficulty in walking	To facilitate mobility	Gait training	Gait training with visual and verbal cues	15 minutes per day progressed to 30 minutes per day

Follow-up and outcomes

An organized physical therapy intervention protocol was started. For four weeks, a follow-up was carried out once per week. The findings of the outcome measure are shown in Tables 4, 5.

**Table 4:** Pre- and post-physiotherapeutic rehabilitation ROM ROM: range of motion

	Right lower limb		Left lower limb	
Joints	ROM pre intervention	ROM post intervention	ROM pre intervention	ROM post intervention
Hip flexion	40°	100°	95°	110°
Hip abduction	30°	40°	25°	40°
Knee flexion	40°	100°	90°	110°

**Table 5:** Pre- and post-physiotherapeutic rehabilitation MMT according to MRC grading of the right lower limb MMT: manual muscle testing; MRC: Medical Research Council; Grade 0: no contraction; Grade 1: flicker of contraction; Grade 2: full range of motion actively in anti-gravity position; Grade 3: full range of motion actively against gravity; Grade 4: full range of motion actively against gravity with minimal resistance; Grade 5: full range of motion actively against gravity with maximal resistance

Muscles	Pre intervention	Post intervention
Hip flexors	1/5	4/5
Hip extensors	1/5	4/5
Hip abductors	1/5	4/5
Hip adductors	1/5	4/5
Knee flexors	1/5	4/5
Knee extensors	1/5	4/5

## Discussion

In a clinical setting, cryotherapy involves applying cold to the injured area using ice packs or cooled water. It reduces enzyme activity and constricts blood vessels, affecting tissue metabolism, local blood flow, and nerve signal conduction [13]. This reduction in blood flow helps control inflammation, prevent swelling, and minimize blood loss after an injury [14]. The use of a cooling therapy offers benefits that are likely due to a decrease in the inflammatory response, as well as reduced fluid secretion and bleeding [15].

Static strengthening exercises are often used to build and maintain muscle strength, improve stability, and enhance endurance in a specific position or posture. These exercises are in contrast to dynamic exercises, where the joint angle changes during the muscle contraction [16]. As a result of task-specific training, a significant proportion of patients observed enhancements in their capacity to engage in various activities and regain functionality in their daily lives. Over the long term, both function and the ability to perform activities are substantially restored [17]. Patellar mobilization is a technique to improve patella bone mobility, enhancing knee function and reducing pain. It is usually done in the open-packed position of knee extension but can be adapted to more symptomatic positions. The therapist chooses the mobilization direction based on symptom relief or perceived restrictions, using medial/lateral glides for knee rotation and patellofemoral glides for joint pain or reduced mobility [18].

Gait training is important post-surgically and often starts with partial weight-bearing, using crutches or other assistive devices [19]. The gradual transition from non-weight-bearing to partial and then full weight-bearing is essential to prevent excessive stress on the surgically corrected area. Patients may use crutches, walkers, or canes to aid in maintaining balance and stability while they regain confidence in walking with their fixed alignment [20].

## Conclusions

PT, followed after orthopaedic management, incorporates a tailored treatment regimen and assumes a pivotal role in the recuperation of patients who have undergone soft tissue reconstruction and hemiepiphysiodesis. This method has the potential to yield significant enhancements in muscular strength and holistic functionality, thereby fostering superior patient results.
